# Disparate Entry of Adenoviruses Dictates Differential Innate Immune Responses on the Ocular Surface

**DOI:** 10.3390/microorganisms7090351

**Published:** 2019-09-13

**Authors:** Matthew R. Pennington, Amrita Saha, David F. Painter, Christina Gavazzi, Ashrafali M. Ismail, Xiaohong Zhou, James Chodosh, Jaya Rajaiya

**Affiliations:** Howe Laboratory, Massachusetts Eye and Ear, Department of Ophthalmology, Harvard Medical School, Boston, MA 02114, USA; matthew_pennington@meei.harvard.edu (M.R.P.); amrita_saha@meei.harvard.edu (A.S.); david_painter@meei.harvard.edu (D.F.P.); christina_gavazzi@college.harvard.edu (C.G.); mohamed_ismail@meei.harvard.edu (A.M.I.); xiaohong_zhou@meei.harvard.edu (X.Z.); james_chodosh@meei.harvard.edu (J.C.)

**Keywords:** adenovirus, caveolin, clathrin, cornea, dynamin, endocytosis, epidemic keratoconjunctivitis, entry, innate immunity, macropinocytosis, ocular surface, receptors, trafficking

## Abstract

Human adenovirus infection of the ocular surface is associated with severe keratoconjunctivitis and the formation of subepithelial corneal infiltrates, which may persist and impair vision for months to years following infection. Long term pathology persists well beyond the resolution of viral replication, indicating that the prolonged immune response is not virus-mediated. However, it is not clear how these responses are sustained or even initiated following infection. This review discusses recent work from our laboratory and others which demonstrates different entry pathways specific to both adenovirus and cell type. These findings suggest that adenoviruses may stimulate specific pattern recognition receptors in an entry/trafficking-dependent manner, leading to distinct immune responses dependent on the virus/cell type combination. Additional work is needed to understand the specific connections between adenoviral entry and the stimulation of innate immune responses by the various cell types present on the ocular surface.

## 1. Introduction

Conjunctivitis, a common ocular condition with a range of etiologies, is highly prevalent, affecting approximately 6 million people annually in the United States and accounting for 1% of all primary care office visits [1,2,3,4]. Viruses are responsible for up to 80% of conjunctivitis, and human adenoviruses (HAdVs) are implicated in up to 65% of all viral cases [5,6]. Adenoviruses are small, non-enveloped viruses with a linear, double-stranded DNA genome of approximately 36 kilobase pairs. The seven species (A-G) and more than one hundred genotypes currently in GenBank exhibit a broad range of tropisms across the various mucosal surfaces of the body, including those within the respiratory, gastrointestinal, and genitourinary tracts, in addition to cells on the ocular surface. While adenovirus infections are generally acute and self-limiting in immunocompetent patients, they can be fatal in children and immunocompromised individuals [7,8,9].

**Figure 1:**
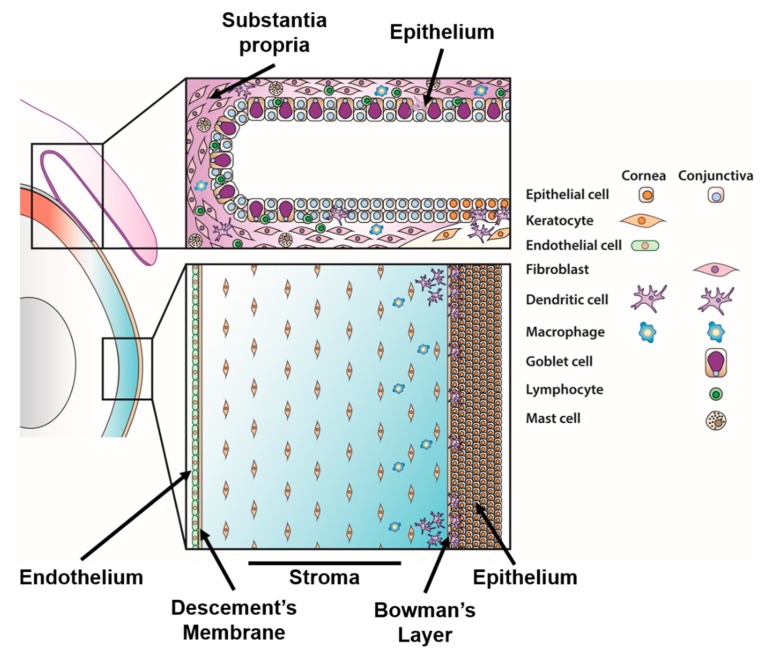
General structure of the normal human ocular surface. Inserts: cellular arrangement of the conjunctiva (upper) and cornea (lower).

The most severe adenovirus infections of the ocular surface are associated with HAdVs of species D (HAdV-D) [10], the species with the largest number of described viruses. The term “ocular surface” broadly refers to the cornea and the conjunctiva (Figure 1). The principal function of the cornea is to refract incoming light to the lens, where the light is then focused onto the retina for visual discrimination. The cornea is composed of five distinct layers (from anterior to posterior): the epithelium, Bowman’s layer, stroma, Descemet’s membrane, and endothelium [11]. The stroma, sometimes called the corneal substantia propria, accounts for approximately 85% of the total corneal thickness and houses a large population of keratocytes and a small number of corneal resident immune cells [12,13]. The conjunctiva is the mucus membrane that lines the inside of the eyelids and covers the globe. It consists of an outer three to five cell layers thick epithelium of stratified squamous and columnar epithelial cells interspersed with goblet cells. It also contains blood vessels, lymphatic channels, and numerous immune cells, including T and dendritic cells. The conjunctival substantia propria is its deeper layer, and is rich in connective tissue, lymphocytes, mast cells, plasma cells, and occasionally neutrophils in the normal conjunctiva [14,15,16].

**Figure 2:**
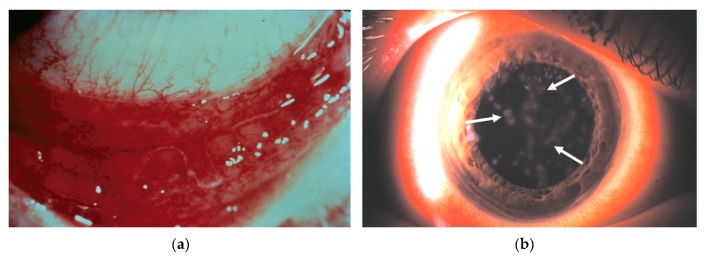
Common clinical manifestations of epidemic keratoconjunctivitis (EKC). (**a**) Photomicrograph of the inferior conjunctival fornix of a patient with acute EKC, showing lymphoid follicles and subconjunctival hemorrhage. (**b**) Photomicrograph of an eye with corneal subepithelial infiltrates (white arrows).

Perhaps due to the relatively self-limiting nature of most conjunctivitis presentations, in which symptoms usually resolve within two to three weeks of onset, very few studies have investigated conjunctival immune responses to virus infection [17]. Clinically, however, ocular HAdV infection generally presents as one of three highly contagious syndromes: follicular conjunctivitis, pharyngoconjunctival fever (PCF), or epidemic keratoconjunctivitis (EKC) [18]. Follicular conjunctivitis is characterized by bulbar conjunctival injection and chemosis, follicular hyperplasia, preauricular adenopathy, and sometimes conjunctival petechiae or frank subconjunctival hemorrhages. PCF appears similar, however, in addition to the ocular signs, is associated with a systemic, flu-like illness [19,20]. EKC, which is most commonly caused by HAdV-D8, -37, -53, -54, -56, and -64, is a severe, hyperacute, and particularly contagious infection [21,22]. EKC is characterized by acute membranous keratoconjunctivitis and delayed-onset subepithelial corneal infiltrates (SEIs) (Figure 2).

SEIs, the hallmark feature of EKC, occur in approximately one-third of all EKC cases and may persist or recur for months to years following infection [23,24,25,26]. SEIs impair vision by physically blocking the passage of light and by disrupting the arrangement of collagen fibrils and other extracellular matrix components of the meticulously organized and normally transparent corneal stroma [27,28,29,30,31,32]. Clinically, this manifests as reduced vision, foreign body sensation, and photophobia [33,34,35]. Based on both experimental and clinical evidence, SEIs form as a consequence of infiltrating leukocytes, recruited from the corneal limbus to the superficial corneal stroma [31,32]. SEI appearance is delayed by up to three weeks from the onset of infection, a time when active viral replication has ceased [36], which suggests that long term morbidity associated with infection is immune-mediated rather than a result of virus-associated tissue damage. Unfortunately, despite frequent outbreaks of adenoviral conjunctivitis and the substantial economic impacts—due to lost work time and expenses associated with medical visits and diagnostic testing—there are currently no specific antiviral therapies for adenovirus ocular infections. Further, due to their apparent immunological origin, SEIs are unresponsive to direct-acting antivirals. The elucidation of the immunopathogenesis of infection and its relationship with the virus replication cycle is crucial to the potential development of future immunomodulatory therapies.

Dogma maintains that HAdVs enter host cells via dynamin-dependent, clathrin-mediated endocytosis before trafficking along microtubules to the nucleus for replication [37,38]. However, recent work has demonstrated that adenoviruses utilize several entry mechanisms, including macropinocytosis [39,40,41] and caveolin-mediated pathways [42]. The specific mechanism of entry appears to depend most on the specific pairing of cell and virus type. In some cell types, viruses may exploit more than one pathway with no apparent preference. Furthermore, a predominant pathway may be supplanted by another pathway if the former is blocked. Redundancy in both entry and subsequent immune responses may be the rule rather than the exception. Furthermore, analyses of viral entry may be complicated by the finding that some host signaling proteins that were initially identified as specific to a particular pathway are in fact shared by disparate pathways.

Innate immune responses to adenoviruses rely on the detection of pathogen-associated molecular patterns (PAMPs): distinct ligands present on the external surfaces, and nucleic acids of pathogens (but absent in the host) that feature molecular signatures able to be recognized by pattern recognition receptors (PRR) on or in infected host cells [43,44,45,46]. Due to the specific distribution of these PRRs on the cell surface, in endosomes, and in the cytosol, it is expected that adenoviruses utilizing disparate entry and trafficking mechanisms may stimulate specific and unique subsets of PRRs, ultimately resulting in unique immune response signatures. Consistent with this hypothesis, it was shown that rapidly trafficking adenoviruses replicate more efficiently [47], but may not stimulate host cytokine responses as effectively as a virus that enters and traffics more slowly [22]. However, such a relationship has yet to be fully defined in the context of ocular surface cells. This review will focus broadly on the mechanisms of adenoviral entry and trafficking, the immune responses to adenovirus infection of the ocular surface, and the possible connection between the two.

## 2. Adenoviral Entry and Trafficking

The HAdV genome is encapsidated by an icosahedral protein shell (capsid) composed of three major capsid proteins (hexon, penton base, and fiber) and four minor/cement proteins (IIIa, VI, VIII, and IX). The hexon protein is the most abundant adenovirus protein, with 240 hexon trimers (720 individual hexon proteins) forming the bulk of the capsid structure. Each of the 12 capsid vertices is formed by a ring of five penton base proteins, from which a trimeric fiber protein protrudes, ending in its distal fiber knob (reviewed in [48]). The infection cycle begins with the binding of the fiber knob to a cell surface receptor. Most HAdVs, except HAdV-B and some members of HAdV-D, utilize the coxsackievirus-adenovirus receptor (CAR), which is expressed in most human tissues [49,50,51]. Typically, HAdV-Bs bind CD46, a complement regulatory protein, as the primary receptor [52]. The EKC-associated viruses also bind CD46 and additionally bind ganglioside sialic acids, notably GD1a [50,53,54,55,56,57,58]. Receptor binding draws the capsid toward the cell membrane, enabling an interaction between an Arginine-Glycine-Aspartic acid (RGD) motif in the outer loop of each penton base protein and cell surface integrins, which serve as secondary receptors. Specifically, for HAdV-D37, it has been shown that αVβ1 and αVβ3 are utilized for the infection of corneal epithelial cells [59,60]. The binding of the RGD motif induces conformational changes in the integrins, which mediate downstream intracellular signaling to promote viral entry into the host cell. It is generally thought that specific fiber knob amino acids and the availability of the necessary cellular receptors and integrins determine the tropism of HAdVs for the ocular surface [59,61]. Indeed, we recently illustrated positive selection pressure on one particular amino acid in the fiber knob, specifically a lysine or alanine at residue 240, which is critical for corneal tropism and differentiates EKC adenoviruses from non-EKC viruses [62]. For non-ocular, CAR-utilizing viruses, expression levels of CAR control entry and nuclear trafficking efficiency (reviewed in [63]). However, it is important to note that entry into a specific cell type does not guarantee successful trafficking or viral replication.

Few studies to date have specifically sought to characterize the entry mechanisms for EKC-associated HAdVs in ocular cells. Most have focused on the non-EKC-associated, species C, specifically HAdV-C2 and -5, as well as species B, HAdV-B3, -7,-9, and -35 [63]. In addition, immortalized human cell lines, including HeLa cells, KB cells (subcloned from HeLa cells), and A549 cells, have been the preferred cell types for HAdV-C2 and -5 entry studies due to their support for rapid and robust viral replication observed in these cells [63]. These studies are frequently cited as support for clathrin-mediated endocytosis as the sole or primary means by which adenoviruses enter cells [10]. Further work with HAdV-Cs has led to the idea that subsequent trafficking to the nucleus occurs along the microtubule network in a dynamin-dependent manner [64,65,66,67]. However, recent experimental evidence supports viral utilization of several entry mechanisms, including clathrin-mediated endocytosis [64], macropinocytosis [39], and caveolin-mediated pathways [42].

### 2.1. Clathrin-mediated Endocytosis

**Figure 3:**
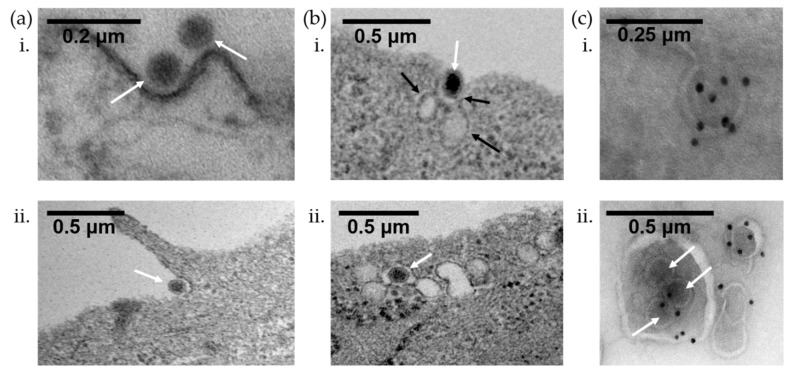
Electron micrographs of HAdV-D37 entering ocular surface cells. Corneal keratocytes or tert-immortalized human corneal epithelial cells were infected with MOI = 10 of cesium chloride purified HAdV-D37 for 1 hour at room temperature. Cells were washed with PBS, fixed in a fixative solution (2% paraformaldehyde containing 2.5% glutaraldehyde, 0.1 M cacodylate, and 2.5 mM CaCl_2_) for 1 hour, and collected in 2% agarose. The cell pellet was further fixed for 1.5 hours in 2% aqueous OsO4 and dehydrated. After dehydration, the cell pellet was embedded in epon and sectioned into 70–90 nm thin sections. The sections were stained with saturated aqueous uranyl acetate, Sato’s lead stain, and micrographs were taken on a Philips CM-10 electron microscope operating at 80 kv and fitted to a CCD camera. (**a**) Electron micrographs of infected tert-immortalized human corneal epithelial cells show that HAdV-D37 (white arrows) can enter via clathrin-mediated endocytosis (i) or macropinocytosis (ii). (**b**) Electron micrographs of infected keratocytes showing caveolae (black arrows) associated with HAdV-D37 (white arrows) at the cell membrane (i) and inside a caveosome (ii). (**c**) Immunogold staining for caveolin in uninfected (i) and HAdV-D37 infected (ii) keratocytes (white arrows indicate virus).

Clathrin-mediated endocytosis is the best elucidated route for viral entry, including for adenovirus [64,68,69,70,71]. This entry process involves the internalization of virions into a double membrane coated pit with triskelion-shaped clathrin proteins, which collectively interact to form a polyhedral lattice surrounding the endocytosed vesicle [64,72,73]. For cornea-tropic adenoviruses, there is a general paucity of data on the entry mechanism. However, in agreement with previous studies, we found that HAdV-D37 infection of immortalized human corneal epithelial cells was predominantly clathrin-mediated (Figure 3a(i)), with a lesser contribution from macropinocytosis (Figure 3a(ii)).

Factors beyond just the clathrin-coated pit are required for clathrin-mediated endocytosis [39]. For example, dynamin, a 100-kDa GTPase, plays a critical role in the fission of newly formed vesicles, without which clathrin-mediated endocytosis does not occur [74]. Of the three dynamin isoforms, dynamin 2 is expressed in most cell types. It also functions as a microtubule binding protein [75] and as a negative regulator of microtubule stability [76]. The binding of dynamin 2 to microtubules activates its GTPase function, resulting in endosome migration along the cytoskeletal network [77]. Clathrin-mediated endocytosis of HAdV-C2 and -5 has been shown to require dynamin activity [37,64,78]. However, dynamin is not required for the entry of all adenoviruses. For example, HAdV-B3 utilizes dynamin-independent endocytosis for rapid entry into epithelial and hematopoietic cells [79].

Adenovirus infection of human corneal epithelial cells is highly restricted [42,80], and in vitro tropism of specific HAdV types for immortalized human corneal epithelial cells very closely matches the known associations between specific HAdV types and clinical infection in EKC [22,81,82]. Corneal epithelial cell infection by tropic HAdVs also occurs via a dynamin 2-independent pathway (manuscript in preparation). Further, in fibroblasts derived from human corneal stromal keratocytes—also known as human corneal fibroblasts (HCFs)—dynamin 2 activity had no impact on the cellular entry of HAdV-D37 but did influence the delivery of viral DNA to cell nuclei. Specifically, the knockdown of dynamin 2 resulted in increased microtubule acetylation, closer proximity of microtubule organizing centers (MTOCs) to cell nuclei, and increased perinuclear HAdV-D37 localization [38]. Consistent with others’ findings that adenovirus accumulation around the MTOC is a prerequisite to nuclear entry [83], dyamin 2 knockdown in corneal fibroblasts resulted in greater delivery of adenoviral DNA into cell nuclei. In parallel experiments, overexpression of dynamin 2 resulted in the opposite effects, including reduced nuclear entry of viral DNA [38]. These data confirm a dichotomous role for dynamin in adenoviral entry, dependent on both cell and virus type.

### 2.2. Caveolin-Mediated Endocytosis

Caveolae-dependent cell entry of adenovirus is controversial. Caveolae are flask- or omega-shaped lipid raft invaginations of the plasma membrane, with an average diameter of 50–100 nm [84,85,86]. Caveloae are abundantly present in many cell types including fibroblasts, cardiomyocytes, and adipocytes, but are not common to all eukaryotic cell membranes [87]. Caveolins are the major integral membrane proteins of caveolae and include three types: caveolin-1 and -2 are found in most cell types, while caveolin-3 is present only in myocytes [88,89]. Caveolae exist stably at the plasma membrane, but following internalization, they fuse with other caveolae to form larger structures called caveosomes, or they fuse with endosomes in a Rab5-dependent manner [90]. While many molecules are needed to initiate the internalization of caveolae, dynamin 2, and Src, PKC activation and recruitment of actin appear to be important [91,92,93,94,95,96]. Caveolae have been implicated in potential entry pathways for a diverse range of viruses, including coronavirus [97], Hepatitis B virus [98], polyomavirus [99], papillomavirus [100], simian virus 40 [101], filoviruses [102], and human immunodeficiency virus [103,104], among others [105,106,107,108,109,110,111].

Our work has indicated that HAdV-D37 enters human keratocytes in vitro using caveolae [42], while the non-cornea tropic HAdV-C2 fails to infect keratocytes altogether. Confocal analysis of infected cells revealed robust colocalization of HAdV-D37 with caveolin-1, but not LAMP1, the latter of which is a late endosomal marker. A general increase in caveolin-1 in lipid rafts as well as increased Src phosphorylation was noted in the infected cells. Caveolin-rich endosomal fractions were found to contain higher levels of viral DNA compared to fractions rich in LAMP1. IL-8, a cytokine rapidly induced following HAdV-D37 infection, was found to be reduced following caveolin-1 knockdown. Electron microscopy (EM) of the infected cells found multiple flask-shaped vesicles resembling caveolae and caveosome-like structures both contain HAdV-D37 virions (Figure 3b). Using immunoelectron microscopy, these virus-containing invaginations were also found to express caveolin-1 (Figure 3c). Utilizing a novel mouse model of infection, caveolin-1 deficient mice were found to accumulate virus on the cell membranes of corneal stromal cells and exhibit delayed viral entry compared to wild type mice. Further, Src phosphorylation and expression of CXCL1 (a murine analog of IL-8) were both reduced in infected, caveolin-1 knockout mice compared to control mice. Collectively, these data support a caveolin-dependent entry pathway for HAdV-D37 in the cornea stromal cells. In contrast, in A549 cells, HAdV-D37 colocalized with LAMP1, which is consistent with an endosomal trafficking pathway in these particular epithelial cells.

### 2.3. Macropinocytosis

Macropinocytosis is an endocytic process that begins with interactions between a ligand and its host cell receptors, activating a cascade of intracellular signaling and actin rearrangement which drives the formation of cell membrane ruffles on the host cell surface. These ruffles enclose the cargo, forming a large (>250 nm) vesicle known as a macropinosome near the plasma membrane, which then releases the cargo into the cytosol [112,113]. Macropinocytosis may either be constitutively active, as in dendritic cells, or induced. Many families of viruses, including herpesviruses [114,115], vaccinia viruses [116,117], picornaviruses [118], and some adenoviruses [39,40,41], are known to exploit macropinocytosis for their entry. While clathrin-mediated endocytosis is the predominant entry pathway for HAdV-C2 and -5, as discussed above, these viruses can simultaneously utilize macropinocytosis as an alternative entry mechanism. Additionally, if clathrin-mediated endocytosis is blocked, macropinocytosis becomes the primary means of entry [39]. In contrast, HAdV-B3 and -35 utilize macropinocytosis as their primary entry pathway. Macropinocytosis is also variably dependent on dynamin activity. HAdV-B3 entry via macropinocytosis does not require dynamin, whereas macropinocytosis of HAdV-B35 requires dynamin for entry into the HeLa-Kyoto clonal derivation, but not into the parental HeLa-ATCC cells or Wi-38 cells, a diploid human fibroblast cell line derived from lung tissue [41,79].

In a tert-immortalized human corneal epithelial cell line, as shown by EM, macropinocytosis was also utilized for adenovirus entry (Figure 3a(ii)). Macropinocytosis is regulated by p21-activated kinase (Pak), a serine/threonine kinase, and pretreatment with the Pak inhibitor IPA-3 reduced viral gene expression in a dose-dependent manner. Interestingly, the sodium-proton exchange inhibitor 5-(N-ethyl-N-isopropyl) amiloride did not block the endocytic uptake of HAdV-D37 (manuscript in preparation). Further, clathrin knockdown completely abrogated viral entry, while macropinocytosis blocking agents did not. This strongly suggests that clathrin-mediated endocytosis is the dominant pathway in corneal epithelial cells, with macropinocytosis playing a secondary role. Additional studies are needed in order to define the molecular entry mechanisms of HAdV-D37 and other EKC-associated adenoviruses into ocular surface cells.

### 2.4. Uncoating and Nuclear Trafficking

Following adenoviral entry into the cell, for viral replication to occur, the viral genome must reach the nucleus. Because intact adenovirions are too large to enter the nucleus through the nuclear pore complex, they must first uncoat to release their genome [119]. Mechanical and chemical forces drive this dynamic, tightly-regulated, and irreversible process (reviewed in [120,121,122,123,124]). For viruses utilizing clathrin-mediated endocytosis, such as HAdV-C2 and -5, uncoating begins with the interaction between the penton base RGD motifs and cellular integrins. Subsequent fiber-shedding exposes protein VI (pVI) and the virion core is endocytosed. Further, pVI, a 22 kDa cement protein, functions as the primary lytic factor for penetration of the endosomal membrane [125]. In contrast, for caveolin-dependent endocytosis and macropinocytosis, the specific adenovirus uncoating pathway is not well-defined. Once in the cytosol, the adenovirus capsid core associates with microtubule-based motors, including dynein and kinesin, and is transported to the nucleus via the MTOC [126,127,128]. Beyond their role in virion transport, dynein and kinesin also exert mechanical forces on the capsid, completing the uncoating process [128]. Finally, the viral core particle binds to the nucleoporin Nup214 at the nuclear membrane, leading to kinesin-1 mediated disassembly and enabling the viral genome to enter the nucleus [129,130,131].

## 3. Adenovirus Trafficking and Innate Immunity

The observation that adenoviral entry, uncoating, and trafficking may vary based on virus and cell type has interesting potential implications for host cell immune responses. All cells are capable, at least to some degree, of stimulating local innate immune and antiviral responses. As discussed above, this is largely mediated through the detection of conserved PAMPs on or in invading pathogens by host cell PRRs, inducing both divergent and convergent signaling cascades and leading to the production of inflammatory mediators [43,44,45,46]. PRRs are known to have specific distributions within each cell, unique to cell type, to facilitate the detection of pathogen components based on their cellular location. For example, Toll-like receptors (TLRs) 1, 2, 4, 5, and 6 are expressed on the cell surface, while TLRs 3, 7, 8, and 9 are expressed on intracellular endosomal membranes [132]. Other PRRs, such as the nucleic acid sensors RIG-I, AIM2, cGAS, and the NLRP3 inflammasome complex, are expressed in the cytosol [133]. Many of these PRRs have been shown to function as sentries to detect adenovirus infection [134]. It follows that the specific pathway of adenoviral entry and trafficking could expose adenoviral PAMPs to different host cell PRRs, inducing cell- and virus-type specific responses.

Studies have sought to elucidate the connections between entry, trafficking, and innate immune responses by infected cells [44,135,136,137]. In A549 cells, HAdV-C5 was shown to move relatively quickly (within 1 hour of infection) from early endosomes to the cell nucleus. In contrast, HAdV-D26 and -B35 remained within late endosomes at 2–6 h post-infection. The persistence of virions in late endosomes in human peripheral blood mononuclear cells (PBMCs) was associated with higher expression of IFNα2, IL-1β, IL-6, MIP-1β, and TNFα. Pre-treatment with inhibitors of endosomal acidification reduced expression of these factors, supporting viral accumulation in late endosomes as a strong stimulator of innate immune responses [135]. The same findings were reproduced in vivo. Vaccination of rhesus macaques with HAdV-D26 and HAdV-B35 was associated with higher serum levels of pro-inflammatory cytokines and chemokines than with HAdV-C5 [136]. In other studies, it was shown that divergent PRR activation in different types of immune cells can lead to a convergent interferon (IFN) response. Plasmacytoid dendritic cells, a minor subset of the monocyte population found in blood, recognize adenoviral DNA in the late endosome in a TLR9 and MyD88-dependent manner, leading to the production of IFNα [43]. In conventional dendritic cells, hepatic Kupffer cells, and peritoneal macrophages, all of which are more prevalent than plasmacytoid dendritic cells, recombinant adenoviral vectors utilized a TLR-independent pathway to detect cytosolic viral DNA and drive IFNα production [44]. While the cytosolic DNA sensor has yet to be defined, it was shown that this pathway requires endosomal escape, signaling via SAPK/JNK and IRF3 [137]. Further work is needed to establish specific connections between viral PAMPs and host sensors during adenoviral infection of ocular surface cells.

## 4. Ocular Immune Response to Adenovirus Infection

While the connection between entry, trafficking, and immune responses has not been specifically addressed in the cell types present on the ocular surface, general immune responses to adenovirus have been studied. Since the 1940s, it has been understood that the cornea is an immune privileged site capable of mounting immune responses that both protect against insult and preserve the anatomical integrity and visual function of the eye (reviewed in [138,139]). As EKC is the ocular adenovirus-associated disease most associated with long term morbidity and vision loss [19,140], it is not surprising that the majority of research over the past 70 years has largely focused on immune responses within the cornea. These studies, discussed below, suggest that different ocular surface cells respond in immunologically distinct ways.

### 4.1. Corneal Immunity

#### 4.1.1. Corneal Epithelial Cell Responses

Primary viral replication in corneal epithelial cells results in punctate and geographic epithelial keratitis [141,142,143,144]. Corneal epithelial cells produce a variety of cytokines, including CCL20, EGF, IL-1α, IL-6, IL-8, and TGF-β1 [145,146,147,148,149], in response to different stimuli, though typically at lower levels relative to other corneal cell types. Few studies have examined the cytokine responses of corneal epithelial cells to ocular adenoviruses. One study showed that IL-1α secreted by HAdV-D37 infected epithelial cells can enhance ICAM-1 expression on endothelial cells to promote lymphocyte entry into the infected cornea, thereby promoting SEI formation [150]. In immortalized corneal epithelial cells, although HAdV-D37 was able to enter and replicate, our attempts to profile cytokine induction using cytokine arrays failed to identify any elevation of commonly studied cytokines (unpublished data). This is consistent with similar studies performed on corneal epithelial cells with human alphaherpesvirus 1, in which chemokine induction by infected cells was meager in comparison to that by infected corneal fibroblasts [146]. Perhaps the study of monolayer epithelial cell cultures in isolation from other cell types, those that are normally present in vivo, is misleading. It is also possible that use of primary corneal epithelial cell cultures and/or a more sensitive cytokine detection methodology would lead to a different conclusion.

#### 4.1.2. Corneal Keratocyte Responses

Since the 1950s, it has become clear that the adenovirus-infected corneal stroma is more than just a bystander or mere target of corneal inflammation, as famously suggested by Barrie Jones [151]. Instead, immune responses to adenovirus infection involve the active participation of stromal resident cells, including keratocytes, which are capable of inducing and orchestrating the secretion of specific immune factors. Keratocytes are the major cell population of the stroma (Figure 1) and play important roles in preserving the clarity and highly ordered structure of the stroma [28,152]. In vitro, cultured keratocytes resemble fibroblasts and have been shown to produce a broad and robust spectrum of cytokines, including CCL11 [153,154], CCL20 [149], CXCL9 [155,156], G-CSF [157], GRO-alpha/CXCL1 [158], IFN-γ [154], IL-1α/β [148,159], IL-6 [147,154,160,161], IL-8 [145,146,153,154,162,163,164,165,166,167], IL-12 [154], IP-10 [154,155], MCP-1/CCL2, RANTES [154,161,163,167,168,169,170], TGF-β1 [148], and TNFα [160]. Additionally, the aforementioned studies found that keratocytes are often more potent producers of pro-inflammatory cytokines than corneal epithelial cells, indicating that they have a considerable role in the orchestration of corneal immune responses. In vitro, adenoviral infected keratocytes express the pro-inflammatory chemokines IL-6, IL-8/CXCL8, and MCP-1/CCL2, which promote subsequent immune cell infiltration to the cornea and SEI formation [31,164,171,172]. However, during natural infection of the eye, it has not yet been demonstrated if adenovirus is capable of reaching the corneal stroma.

**Figure 4:**
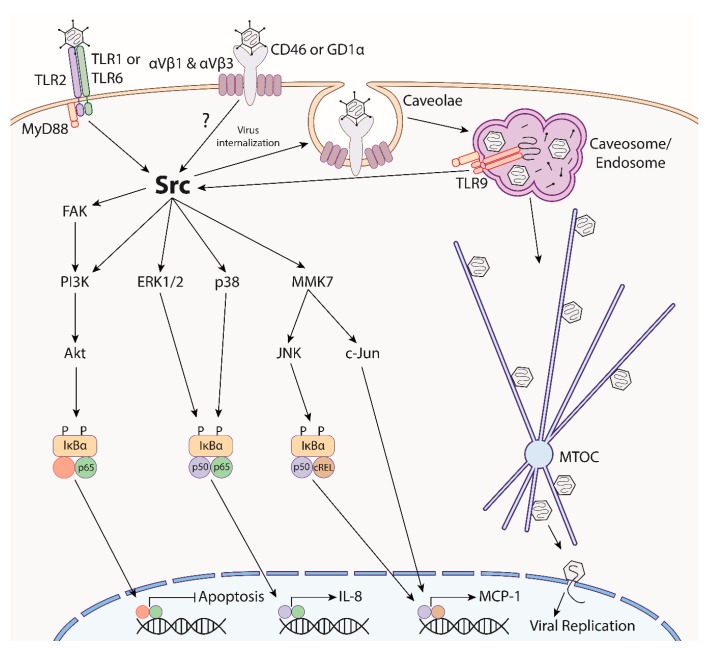
Overview of human adenovirus induced cell signaling and downstream immune responses in human keratocytes, highlighting the centrality of Src kinase. Following engagement with the primary receptor (CD46 or GD1α) by the viral fiber protein and secondary engagement of the penton base with the αVβ1 or αVβ3 integrins, group D adenoviruses are internalized via Src-dependent, caveolin-mediated endocytosis. Following uncoating and fiber shedding, virions traffic along the microtubule network, through the microtubule organizing center (MTOC), and the viral genome enters the nucleus for replication. Adenovirus stimulates cell surface TLR2 and endosomal TLR9, which synergistically activate MyD88. MyD88 further activates Src, which then mediates multiple downstream kinases, leading to NFκB activation. This culminates in the inhibition of apoptosis and expression of pro-inflammatory genes, including IL-8 and MCP-1. A similar signaling pathway in human corneal epithelial cells has yet to be elucidated.

Work from our laboratory has defined the signaling pathways by which these cytokines are stimulated in keratocytes (Figure 4). The PRRs TLR2 and TLR9 were shown to mediate cytokine responses to EKC-associated adenoviruses [172,173]. TLR2 is expressed on the cell surface and may recognize adenoviruses [174,175]. TLR9 is expressed in the endosome and classically detects unmethylated CpG regions of viral DNA [176]. TLR2 and TLR9 act synergistically and the knock-down of both was required to reduce keratitis in a mouse model [173]. However, pro-inflammatory cytokine production, immune cell recruitment, and keratitis, although reduced, were still noted, indicating that there are likely other, as of yet undefined, PRRs responsible for mediating the innate immune responses to adenoviruses in HCFs.

Following the stimulation of TLR2 and TLR9, MyD88 is recruited and initiates a signaling cascade that results in the activation of Src kinase [173], a protein-tyrosine kinase which plays key roles in cell growth, division, migration, and survival pathways (Figure 4) (reviewed in [177]). Src is also a central signaling molecule in keratocytes in response to adenovirus infection [165] and appears to be phosphorylated within minutes of infection by at least two mechanisms. First, MyD88 was shown to physically interact with and phosphorylate Src, suggesting that TLR activation may directly activate this kinase [173]. Second, virus-integrin binding can also lead to Src activation [165]. MyD88 downstream signaling via IRAK1/4 and TRAF6 can also lead to the activation of the same downstream signaling pathways and pro-inflammatory responses as in Src activation [178]. Src promotes a cell survival pathway by activating the p85 subunit of the phosphoinositide 3-kinase (PI3K), which then activates AKT to drive translocation of NFκB p65 to the nucleus. This pathway inhibits caspase 3/7-dependent apoptosis and thereby promotes cell survival, corresponding with increased viral titers, presumably due to prolongation of cell viability [179]. Src also activates focal adhesion kinase (FAK) within 15 minutes of infection, leading to PI3K activation. It is not known if PI3K activation is dependent on FAK or is directly activated by Src [180].

Src activation induces the expression of IL-8 and MCP-1 by activating the mitogen-activated kinases (MAPKs), including p38, JNK, and ERK1/2 [165]. This results in translocation of the p65 and p50 subunits of NFκB to the nucleus, with subsequent binding to and transcriptional activation of the IL-8 promoter [181]. Src kinase phosphorylates the p38 MAPK kinase, leading to IκB phosphorylation, with convergence to the same pathway as ERK1/2, resulting in p65/p50 translocation and IL-8 expression within 1 hour post-infection [182]. Src also phosphorylates MMK7 shortly after infection, leading to JNK and c-Jun activation, and subsequent MCP-1 expression [183]. Inhibitors of JNK decrease cREL protein binding to MCP-1 promoters and reduce MCP-1 expression at 4 hours post-infection [181]. IL-8 and MCP-1 are therefore mediated by two distinct pathways, explaining why MCP-1 expression occurs later in infection compared to IL-8 [181,182].

#### 4.1.3. Formation of Subepithelial Infiltrates

Animal and human clinical studies have shown that SEIs form in the superficial stroma just below Bowman’s layer and including the corneal epithelial basement membrane (Figure 1), areas which are comprised of a variety of immune cells. Acutely, SEI are comprised of polymorphonuclear leukocytes, but T lymphocytes and dendritic cells have been identified at later time points [24,25,29,30,32,184,185]. Neutrophils are the first and by far the most abundant cell type recruited to the cornea, typically within the first 24 hours post-infection in the mouse model, and are a critical pathogenic event in this infection [31,32,172,181]. IL-8 is a well-described and highly potent neutrophil chemoattractant and its expression in the adenovirus infected cornea closely correlates to the rapid infiltration of neutrophils [164,186]. In a three-dimensional culture system incorporating primary corneal fibroblasts and extracellular matrix, we were able to mimic human corneal infection by adenoviruses. IL-8 was produced by virus infected stromal cells within the cornea facsimile and was bound to heparin sulfate in the facsimile basement membrane, thus presenting a reservoir of chemoattractant for subsequent leukocyte infiltration [187]. Similarly, MCP-1 is a chemoattractant for inflammatory monocytes [188] and is thought to be the chemokine responsible for the delayed migration of monocytes in the adenovirus infected cornea [31]. Keratocytes are also known to upregulate adhesion molecules following infection, including ICAM-1/CD54, which are hypothesized to further promote the extravasation of leukocytes into the cornea [165,167,189]. In addition, as mentioned earlier, corneal epithelial cell-derived interleukin-1 alpha (IL-1α) promotes the expression of ICAM-1 and VCAM-1 on endothelial cells following infection. This allows for transendothelial leukocyte migration and recruitment to the infected cornea [150]. The combination of these cytokines promotes a rapid infiltration of immune cells to the cornea.

Studies evaluating the effects of corticosteroid treatment on corneal SEIs have shown that immune cell infiltrate/SEI formation is reduced, however both the duration and magnitude of virus shedding are increased [190,191,192,193,194]. These data suggest that the immune response also serves to limit viral replication. Paradoxically, keratitis does not appear to require active viral replication. In the mouse model, UV-inactivated virus was found to be sufficient to induce keratitis, while heat denatured capsid was unable to induce keratitis. This study showed that HAdV-D37 viral DNA played only a minor role in innate immune responses, while implicating capsid penton base RGD in the onset of clinical keratitis. In further support of this finding, injection of empty capsid directly into the stroma provoked similar cytokine responses, leukocyte infiltration, and clinical disease as intact virus [172]. Unadulterated adenovirus does not replicate in the mouse cornea, further supporting the notion that intact and replicating virus is not required for inflammation [32,195]. It is not known why SEIs can persist and/or recur for months to years following infection. Their presence does not appear to be mediated by viral antigens because, years after infection, adenoviral particles are not apparent by EM [184] and adenoviral antigen is not detected by immunofluorescence [185].

Other PRRs, viral ligands, and cytokines are likely critical to corneal inflammation after adenoviral infection. For example, microarray analysis of HAdV-64 infected keratocytes showed upregulation of genes related to cell growth and differentiation, apoptosis and oncogenesis, cell signaling, transcription, and immune responses, including FRA1, G-β, LIF, MIP-2α, thymosin beta 4, and IL-8, among others [165,196]. Microarray analysis and transcriptome sequencing of non-ocular cell types infected with different adenovirus types have defined robust changes in innate immune responses. These included the TLR signaling, cell cycle progression, apoptotic, RNA binding and processing, and NFκB signaling pathways [197,198,199,200]. It is not known if other cell types present on the ocular surface respond to adenovirus infection in a similar fashion.

#### 4.1.4. Corneal Resident Immune Cells

Healthy, uninfected corneas contain several distinct resident populations of antigen presenting cells (APCs). Conventional dendritic cells (DCs) and possibly also plasmacytoid DCs are present at the level of the corneal epithelium/basement membrane [13]. Macrophages are also found in the anterior corneal stroma [12,13,201]. The limbus and corneal periphery contain mature and immature Langerhans cells (Figure 1) [202]. These resident cells stimulate the recruitment of systemic innate (neutrophil, macrophage, and natural killer cells) and adaptive (T-cells) immune responses to the eye [201,203,204,205,206,207].

Few studies on the role of ocular surface APCs during adenovirus infection have been performed. We recently demonstrated the involvement of myeloid-derived cells in infection using the Macrophage Fas-Induced Apoptosis (MaFIA) mouse. This mouse expresses a membrane bound suicide protein under the control of the myeloid-lineage specific *c-fms* promoter. Following treatment with the FK506 dimerizer AP20187, myeloid cells, including those in the cornea, then undergo apoptosis [208]. AP20187 treated mice were found to have clinically normal corneas; however, following HAdV-D37 infection and in comparison to control and vehicle treated mice, AP20187 treated mice showed reduced immune cell infiltration and reduced myeloperoxidase expression, the latter if which is a correlate for neutrophil infiltration [209]. These data suggest that DCs and macrophages are critical in the development of the immunopathology of keratitis following adenovirus infection. Despite strong evidence for the role of corneal stromal cells in the pathogenesis of adenovirus keratitis in the mouse model, an understanding of their participation in human adenovirus keratitis remains incomplete.

### 4.2. Conjunctival Immunity

The conjunctiva (Figure 1) plays critical roles in protecting the ocular surface from infection. It provides antimicrobial protection and lubrication to the ocular surface via the production of tears and mucins, which collectively form the 3 μm thick tear film. The tear film consists of three layers, an innermost mucin layer, consisting of epithelial cell and conjunctival goblet cell-secreted mucins, an aqueous layer secreted by the lacrimal and accessory lacrimal glands, and an outermost lipid layer, secreted by meibomian glands at the eyelid margin. The aqueous and mucin layers mix in a gradient, with the greatest mucin concentration at the epithelial surface and the greatest aqueous concentration just posterior to the lipid layer. The tear film provides an effective chemical and physical barrier due to mucins, lysozymes, soluble IgA, and antimicrobial peptides that can entrap and/or destroy invading pathogens, though these functions have primarily been studied for bacteria [210]. As discussed earlier, the conjunctiva also houses a variety of immune cells that are capable of responding rapidly to insults on the ocular surface. The conjunctiva appears to be more susceptible to adenovirus infection than the cornea—many adenoviruses cause conjunctivitis, while only a limited number cause keratitis. Additionally, in EKC, conjunctivitis typically precedes keratitis.

#### 4.2.1. Infection of the Conjunctiva

The evidence is clear that ocular-tropic adenoviruses can infect and replicate in the cells of the human conjunctiva. Both fully infectious adenovirus and viral DNA are frequently isolated from conjunctival scrapings during human outbreaks [211,212,213,214]. One report showed the persistence of the virus in the tear film and conjunctiva of a subset of patients for up to a decade following primary infection [215], although the site of viral persistence was not established. Interestingly, studies using a rabbit model of human adenovirus infection have demonstrated that HAdV-C5 is able to infect and replicate in the acinar epithelial cells of the lacrimal gland, possibly contributing to its detection in the tear film [216]. HAdV-D37 replicates efficiently in the Chang C conjunctival cell line in vitro [61], although it is now known that this cell line was established via HeLa contamination, based on isoenzyme analysis, HeLa marker chromosomes, and DNA fingerprinting [217].

A few studies have focused on the conjunctival immune responses to adenovirus infection. It was shown by oligonucleotide microarray analysis that the infection of primary human conjunctival epithelial cells with low multiplicities of infection of HAdV-C5 resulted in the upregulation of CXCL2, CXCL5, CXCL10, CXCL11, and several interferon induced signaling molecules, including IRF7 and STAT1. This suggests that the infection of conjunctival epithelial cells with adenovirus initiates signaling that would be expected to drive the recruitment of neutrophils, monocytes/macrophages, T cells, NK cells, and dendritic cells to the site of infection [218]. However, these experiments were only performed with HAdV-C5, a respiratory virus, and, as discussed, these results cannot be extrapolated to infection by EKC-associated adenoviruses. Nevertheless, in EKC, immune rich conjunctival membranes form in response to adenoviral induced inflammation. These membranes contain macrophages, neutrophils, CD4+ and CD8+ T cells, B cells, Langerhans cells, and activated dendritic cells, in proportion to the degree and intensity of the inflammation [219]. Conjunctival membranes seen in EKC also contain intact adenoviruses and are therefore infectious, as with other ocular secretions in EKC. In the microarray study mentioned above [218], in addition to their participation in recruiting leukocytes during inflammation, an anti-microbial peptide (defensin)-like role for CXCL10 and CXCL11 was demonstrated against HAdV-B3 and HAdV-C5, but not against HAdV-D8 or HAdV-D64. Likewise, the β-defensins were found to inhibit respiratory, but not ocular genotypes. It has been suggested that ocular-tropic adenovirus types have evolved immune evasion strategies to avoid host defensins, which are abundantly expressed on the ocular surface [220].

#### 4.2.2. Natural Killer Cells

In adenoviral conjunctivitis, it was previously shown that the number of conjunctival lymphocytes, natural killer (NK) cells, and monocytes increases significantly during the acute phase of the infection [221]. Generally, NK cells respond rapidly to viral infection by killing virus infected cells and producing cytokines which promote crosstalk between dendritic cells and T cells, thereby inducing T cell differentiation (reviewed in [222]). Mature CD56^dim^ NK cells that are capable of producing IFNγ are present in the epithelium of healthy conjunctiva [221]. However, upon infection with HAdV-D types, mature NK cells are replaced by CD56^Bright^, immature NK cells. This correlates with the detection of CCL2, CCL3, CCL4, CCL5, CXCL9, and CXCL10 in the tear film, all of which are potent chemokines for immature NK cells. The upregulation of the inhibitory ligand human leukocyte antigen-E on infected epithelial cells and the overall impairment of NK cell responses upon infection with HAdV-D types by reducing the expression of activating ligands on the surface of infected epithelial cells) represent additional mechanisms by which HAdV-D types actively promote immune escape. Notably, non-species D adenoviruses, such as HAdV-C3, -E4, and -C5, were unable to induce these responses. This dampening of antiviral responses likely enables enhanced virus replication in the conjunctiva and, thus, subsequent spread to other ocular surface cells [221].

#### 4.2.3. Conjunctival Mucins and Conjunctival Goblet Cells

Conjunctival epithelial cells express the membrane-associated mucins MUC1, MUC4, and MUC16, which form the protective glycocalyx [223]. Mucins are high molecular weight, heavily glycosylated proteins that have been shown to prevent the penetrance of invading pathogens to the eye. Further, ocular surface mucins have complex O- and N-glycans with sialyated cores [224]. As mentioned, sialic acid residues on the GD1α ganglioside are one of the primary receptors for cornea-tropic HAdV-Ds [53,56]. Therefore, sialic acid containing mucins in the tear film and glycocalyx might act as decoy receptors to reduce subsequent infection of ocular surface cells. While sialic acid-based decoy receptors have been evaluated as a therapy for adenovirus infection [225,226], the potential for sialic acids present in the normal tear film to limit natural infection has not been evaluated to our knowledge. A study from our laboratory found that HAdV-D37 was able to induce ectodomain release of MUC16, resulting in decreased glycocalyx barrier function in cultured corneal and conjunctival epithelial cells. In contrast, HAdV-D19, which is not associated with EKC, was unable to cleave MUC16. This suggests that specific HAdV-D types have evolved in their capacity to penetrate the mucin layer to infect the eye.

Conjunctival goblet cells are another potential mediator of ocular surface immune responses. Goblet cells are important for proper tissue homeostasis through the secretion of the major gel-forming mucin MUC5AC, but they also produce cytokines [227,228]. In response to *Staphylococcus aureus* infection, conjunctival goblet cells produce both mucins and IL-1β, the latter of which is dependent on the NLRP3 inflammasome [229]. Intriguingly, these cells appear to distinguish between commensal and non-toxigenic bacteria, the latter of which does not induce conjunctival goblet cells to initiate an inflammatory response [230]. Paradoxically, it was recently shown that the respiratory pathogen HAdV-C5, but not the enteric pathogen HAdV-F41, preferentially infects goblet cells in human enteroid cultures, suggesting type-specific tropisms for intestinal goblet cells [231]. However, such type-specific tropism and associated immune responses have yet to be investigated for conjunctival goblet cells.

## 5. Future Perspectives

Dogma maintains that adenoviruses enter host cells by clathrin-mediated endocytosis, uncoat within endosomes, and rapidly traffic to the nucleus for subsequent replication. However, this paradigm was established based on studies utilizing a limited number of virus types and using tumor-derived cell lines. Recent work has demonstrated that entry may not always be this straightforward, with adenoviruses entering via diverse and overlapping mechanisms, depending on cell and virus type pairing. Due to the cell-specific and varied pattern of the expression of host PRRs, divergent entry mechanisms have the potential to lead to the stimulation of immune responses that are distinguishable based on the specific cell and virus pair. Such a connection has not been studied for ocular surface cells. We have demonstrated that EKC-associated HAdVs enter and traffic differently in corneal epithelial cells than in stromal keratocytes. Furthermore, HAdVs are known to induce a more diverse and robust cytokine response in stromal keratocytes than in epithelial cells. However, substantial work remains to support or refute the hypothesis that differential entry of adenoviruses to ocular surface cells dictates subsequent immune responses. First, the entry mechanisms of EKC-associated HAdVs would need to be defined across a multitude of cell types, including corneal epithelial cells, keratocytes, conjunctival epithelial cells, conjunctival goblet cells, conjunctival fibroblasts, dendritic cells, macrophages, and other immune cell types that are present on the normal ocular surface. It will be important to determine whether ocular-tropic HAdVs can productively replicate in these cells. Then, the specific immune responses to infection with HAdV will need to be defined, along with the connection between cytokine production and entry pathways. Such experiments would greatly increase our knowledge of ocular surface immunology and may permit the development of therapies that manipulate immune responses to infection to improve the clinical outcomes of infection.
